# Intrapulmonary Gallstones and Pleuro-Biliary Fistula Formation Due to Complication From Prior Cholecystectomy: A Case Report and Literature Review

**DOI:** 10.7759/cureus.25836

**Published:** 2022-06-10

**Authors:** Kyaw Min Tun, Yassin Naga, Jose Aponte-pieras, Bhavana Bhaya

**Affiliations:** 1 Internal Medicine, Kirk Kerkorian School of Medicine at the University of Nevada, Las Vegas, USA; 2 Gastroenterology, Kirk Kerkorian School of Medicine at the University of Nevada, Las Vegas, USA; 3 Internal Medicine, Veterans Affairs (VA) Southern Nevada Healthcare System, Las Vegas, USA

**Keywords:** surgical complications, hemoptysis, pneumobiliary fistula, intrapulmonary gallstones, cholecystectomy

## Abstract

Intrapulmonary gallstones and the formation of pleuro-biliary fistula is a rare complication of laparoscopic cholecystectomy. The stones are most commonly found in the right lower lobe of the lungs. The symptoms tend to be insidious in nature and can manifest as hemoptysis, irritating cough, and cholelithoptysis years after the procedure. The stones can be removed through lobectomy or may also be treated non-invasively with antibiotics only. Here, we describe a case of a patient who developed hemoptysis and was found to have intrapulmonary gallstones from laparoscopic cholecystectomy and subsequent fistula formation.

## Introduction

Cholecystectomy is the standard treatment for cholecystitis and other diseases of the biliary system [1]. Open cholecystectomy was largely replaced by laparoscopic cholecystectomy in 1991 due to increased patient comfort, lower rate of complications, and shorter hospital stays [2]. Complication rates are also higher in open cholecystectomies compared to laparoscopic cholecystectomies and are seen in 16% and 9% of cases, respectively [1]. Laparoscopic cholecystectomy is a safe procedure with a low rate of complication but can cause gallbladder perforation, bile duct injury, and gallstone spillage [3,4]. Transdiaphragmatic migration of spilled gallstones and subsequent formation of a bronchobiliary fistula is a very rare complication from laparoscopic cholecystectomy. Only a few cases have been reported in the literature. Herein, we report a case of an intrapulmonary gallstone in a patient with a remote history of cholecystectomy.

This article was previously presented as a meeting abstract at the 2021 Annual Meeting of the American College of Gastroenterology (ACG) on October 24, 2021, in Las Vegas, Nevada. Neither the full case report nor the literature review has previously been published as a manuscript.

## Case presentation

**Figure 1:**
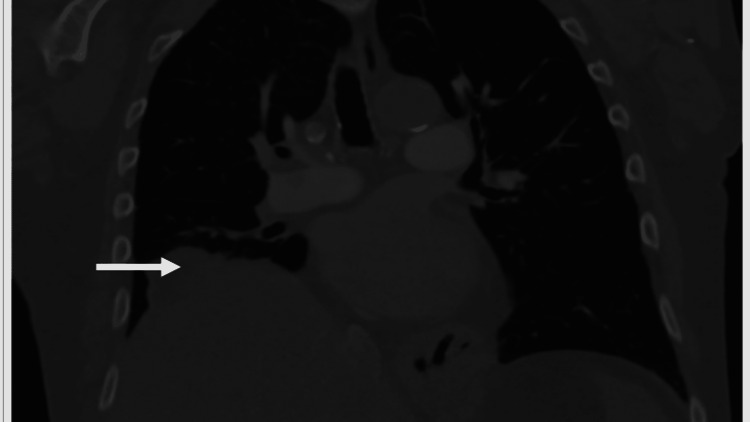
CT angiogram of the thorax illustrating the irregular appearance of the liver dome that was inseparable from the right lower lobe of the lungs (arrow). CT: computerized tomography

**Figure 2:**
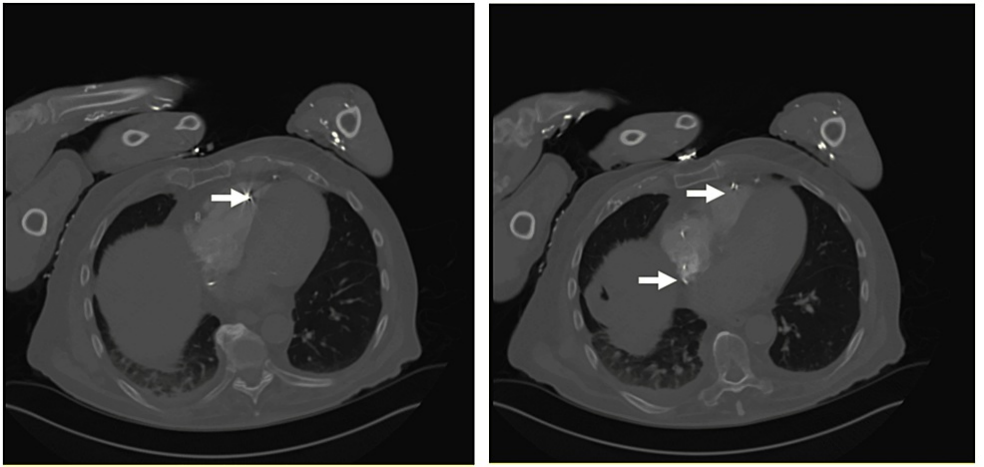
A panel of CT images demonstrates hyper-attenuating structures within the right lower lobe cavity, suggestive of gallstones. CT: computerized tomography

A 90-year-old male with a remote history of a cholecystectomy presented with gangrene of his right third toe and was found to have methicillin-resistant *Staphylococcus aureus* (MRSA) bacteremia. Other pertinent medical history included coronary artery disease with prior percutaneous coronary intervention, pacemaker placement due to complete heart block, Parkinson’s disease, and severe peripheral vascular disease. His gangrenous toe was amputated, and he was started on vancomycin to treat the bacteremia. On postoperative day three, the patient developed an episode of hemoptysis. Given his recent surgery, a computerized tomography (CT) angiogram of the thorax was obtained due to high suspicion of pulmonary embolism. Imaging revealed no evidence of pulmonary embolism. However, cavitation was noted at the right lower lobe of the lungs. Surrounding ground-glass densities and bronchial wall thickening were also noted, suggestive of inflammatory etiology. Furthermore, the region of the cavitation was inseparable from the right hemidiaphragm and the liver dome (Figure 1), and contained ill-defined, mildly hyperattenuating structures that were indicative of displaced gallstones (Figure 2).

The patient and his family reported that a cholecystectomy was performed in the late 1990s. However, the patient’s health records were not available prior to 2010 and thus, the operative report for the cholecystectomy was not available. On physical examination, there was no evidence of a post-surgical scar on the abdomen. Therefore, it was concluded that the procedure was likely laparoscopic rather than open. After the initial episode of hemoptysis in the setting of prophylactic anticoagulation, there was no further recurrence of hemoptysis. There was also no evidence of irritating cough, pleuritic chest pain, or fever. Aminotransaminases, alkaline phosphatase, and total bilirubin were within the reference range during the patient’s hospital course. Given his age, medical comorbidities, and minimal pulmonary symptoms, the patient was determined to be a poor surgical candidate and did not receive any surgical intervention. On hospital day 14, he was discharged to a skilled nursing facility on vancomycin for treatment of the MRSA bacteremia. Approximately two weeks after discharge, the patient underwent septic shock due to persistent MRSA bacteremia and subsequently expired.

## Discussion

Laparoscopic cholecystectomy is generally a safe procedure and has become the standard treatment for cholecystitis and other gallbladder diseases [2]. Although rare, serious thoracic complications can still arise from laparoscopic cholecystectomy, some instances of which include gallbladder perforation and intrapulmonary gallstones [5]. Gallbladder perforation rate is higher in laparoscopic cholecystectomy and is seen in 0.2-20% of cases [3]. Gallstone spillage can occur when the fundus of gallbladder is inadvertently ruptured during the extraction of the organ [6-8]. In fact, stone spillage is not infrequent; it has been observed in 20% of 450 laparoscopic cholecystectomies in a study by Lee et al. [8]. Although the spilled gallstones and bile are usually retrieved, complete removal of all gallstones from the peritoneal cavity is not always possible [5,6,8]. Only 0.3% of stone spillage results in adverse outcomes, including infection, abscess formation, and on rare occasions, pleuro-biliary and broncho-biliary fistula formations [3,5]. The first instance of these fistulas was first reported in 1955 [5].

There are three ways by which gallstones can migrate to the thoracic cavity - through lymphatic channels of Ranvier, congenital diaphragmatic defects, or transdiaphragmatic tracts that result from local infection and inflammation [3]. As seen in our patient’s case, intraperitoneal gallstones can lead to an inflammatory cascade that can erode through the subdiaphragmatic space, causing the formation of a fistula between the biliary tree and the bronchial tree or pleural space [8,9]. The formation of broncho-biliary or pleuro-biliary fistula can lead to a wide array of symptoms including biliptysis, hemoptysis, broncholithiasis, fever, cough, and cholelithoptysis [5,7,9]. Symptom onset can be insidious and can occur several years after the procedure [5].

Due to the scarcity of the cases, there have only been a small number of cases documented in literature. The first incidence of intrathoracic gallstones was published in 1975. Since then, there have only been 18 cases reported and we have compiled them in Table 1.

**Table 1:** Compilation of cases with intrathoracic gallstones with demographic data and treatment outcomes. *Patient was discharged awaiting possible surgical intervention after consultation with general and thoracic surgery teams. **The report only stated that the symptom onset was several months following an elective laparoscopic cholecystectomy. ***Neither full article nor abstract was available despite search from multiple websites and databases. The information is inferred from prior case compilation by Zhang et al. [19]. F: female; M: male; RML: right middle lobe; RLL: right lower lobe; MRSA: methicillin-resistant *Staphylococcus aureus*

Investigator	Presenting symptom	Age (years) and gender	Onset (months)	Location	Treatment	Outcome
Schwegler and Endrei, 1975 [10]	Hemoptysis	64 F	36	RLL	RLL lobectomy	Resolution
Lee et al., 1993 [8]	Massive hemoptysis	58 F	8	RLL	Laparotomy/bronchoscopy	Resolution
Lee et al., 1993 [8]	Cholelithoptysis	52 M	9	RLL	Lung wedge resection	Resolution
Downie et al., 1993 [11]	Cholelithoptysis/hemoptysis	59 F	12	RLL	Bronchoscopy/antibiotics	Unknown*
Thompson et al., 1995 [12]	Cholelithoptysis/hemoptysis	59 F	Unspecified**	RLL	Bronchoscopy/antibiotics/exploratory laparotomy	Resolution
Barnard et al., 1995 [4]	Cholelithoptysis/hemoptysis	54 F	13	RML	Antibiotics/RML lobectomy	Resolution
Breslin and Wadhwa, 1996 [13]	Cholelithoptysis/hemoptysis	54 M	2	RLL	Antibiotics	Resolution
Chan et al., 1998 [14]	Cholelithoptysis	75 F	6	RLL	Antibiotics	Resolution
Baldo et al., 1998 [15]	Cholelithoptysis/hemoptysis	Unknown***	60	RLL	Spontaneous resolution	Resolution
Chopra et al., 1999 [16]	Cholelithoptysis	64 F	30	RLL	Bronchoscopy/antibiotics	Resolution
Werber and Wright, 2001 [6]	Abscess with massive hemoptysis	64 F	6	RLL	RLL wedge resection	Resolution
Houghton et al., 2005 [17]	Cholelithoptysis/hemoptysis	61 F	42	RLL	RLL wedge resection	Resolution
Fontaine et al., 2006 [3]	Hemoptysis	73 F	34	RLL	RLL wedge resection	Resolution
Quail et al., 2014 [18]	Cholelithoptysis/hemoptysis	66 F	60	RLL	RLL wedge resection	Resolution
Zhang et al., 2014 [19]	Cholelithoptysis/hemoptysis	57 M	4	RLL	RLL wedge resection	Resolution
Jones et al., 2015 [5]	Hemoptysis	84 F	5	RLL	RLL wedge resection	Resolution
Binmahfouz and Steinke, 2016 [20]	Massive hemoptysis	66 F	36	RLL	RLL wedge resection	Resolution
This case	Hemoptysis	90 M	240	RLL	Monitoring	Death from sepsis due to MRSA bacteremia

The mean age of patients was 65 years. Symptoms can begin from as few as two months to as long as 20 years since cholecystectomy. Average time of onset of symptoms from cholecystectomy was 35 months. Approximately 72% (13/18) of patients were females while 22% (4/18) were males. Neither the full report nor the abstract by Baldo et al. was available despite extensive literature search; as such, the demographic information was unavailable and the remaining information was inferred from prior compilation by Zhang et al. [15,19].

Management of intrapulmonary gallstones included surgical treatment, such as lobectomy or wedge resection. Patients can also be treated with antibiotics with or without procedural interventions such as bronchoscopy. It has been reported that more invasive measures were employed in patients with recurrent symptoms while non-surgical treatments such as antibiotics were more commonly given in patients with mild to no obvious clinical symptoms [19]. In some cases, intrapulmonary gallstones resolve spontaneously. Among the 18 cases of intrapulmonary gallstones compiled, all but one were found in the right lower lobe, similar to the patient presented. Fistulas may be treated with endoscopic retrograde biliary drainage, and if there is an abscess, thoracotomy or chest tube placement may be performed [6,9]. The duration of time between diagnosis or onset of symptoms and cholecystectomy does not appear to be associated with the need for surgery or adverse outcomes. For instance, the case by Baldo et al. was diagnosed five years after cholecystectomy and resolved spontaneously without treatment [15]. On the other hand, Zhang et al. reported a case where symptoms started four months after the cholecystectomy but required resection of right lower lobe of the lung [19]. Fourteen of 18 cases (78%) were treated with procedural interventions, while four cases (22%) were provided with non-invasive modalities only. Resolution of the symptoms and/or the intrathoracic gallstones was achieved in all cases regardless of the modality of treatment, except in our patient who expired from sepsis due to persistent MRSA bacteremia.

In our patient’s case, given his age, comorbidities, and lack of recurrence of symptoms, a decision was made to not proceed with procedural intervention after discussion of risks and benefits between the medical team and the patient. Although a pulmonary source could not definitely be excluded, the source of bacteremia in our patient was likely from the gangrenous toe. The bacteremia persisted despite treatment and eventually led to sepsis.

## Conclusions

While laparoscopic cholecystectomy is generally a safe procedure, complications can arise from gallstone spillage. Preventative measures include utilization of a retrieval bag to place the resected gallbladder in order to help reduce the incidence of “dropped” gallstones. Our case demonstrates an incidence of both pulmonary gallstones and pleuro-biliary fistula - a rare complication resulting from laparoscopic cholecystectomy that is sparsely documented in literature.
